# Multifunctional Biodegradable Vascular PLLA Scaffold with Improved X-ray Opacity, Anti-Inflammation, and Re-Endothelization

**DOI:** 10.3390/polym13121979

**Published:** 2021-06-16

**Authors:** Ho In Lee, Yun Heo, Seung-Woon Baek, Da-Seul Kim, Duck Hyun Song, Dong Keun Han

**Affiliations:** 1 Department of Biomedical Science, CHA University, 335 Pangyo-ro, Bundang-gu, Seongnam-si 13488, Korea; 016rhk@naver.com (H.I.L.); yheo@chauniv.ac.kr (Y.H.); baiksw830@g.skku.edu (S.-W.B.); kim_daseul@hanmail.net (D.-S.K.); mondh920@naver.com (D.H.S.); 2 Department of Biomedical Engineering, SKKU Institute for Convergence, Sungkyunkwan University (SKKU), 2066 Seobu-ro, Jangan-gu, Suwon-si 16419, Korea; 3 Department of Intelligent Precision Healthcare Convergence, SKKU Institute for Convergence, Sungkyunkwan University (SKKU), 2066 Seobu-ro, Jangan-gu, Suwon-si 16419, Korea; 4 School of Integrative Engineering, Chung-Ang University, 84 Heukseok-ro, Dongjak-gu, Seoul 06974, Korea

**Keywords:** poly(L-lactic acid), magnesium hydroxide, 2,3,5-triiodobenzoic acid, X-ray opacity, anti-inflammation, re-endothelization

## Abstract

Poly(L-lactic acid) (PLLA) has been used as a biodegradable vascular scaffold (BVS) material due to high mechanical property, biodegradability, and biocompatibility. However, acidic byproducts from hydrolysis of PLLA reduce the pH after the surrounding implanted area and cause inflammatory responses. As a result, severe inflammation, thrombosis, and in-stent restenosis can occur after implantation by using BVS. Additionally, polymers such as PLLA could not find on X-ray computed tomography (CT) because of low radiopacity. To this end, here, we fabricated PLLA films as the surface of BVS and divided PLLA films into two coating layers. At the first layer, PLLA film was coated by 2,3,5-triiodobenzoic acid (TIBA) and magnesium hydroxide (MH) with poly(D,L-lactic acid) (PDLLA) for radiopaque and neutralization of acidic environment, respectively. The second layer of coated PLLA films is composed of polydopamine (PDA) and then cystamine (Cys) for the generation of nitric oxide (NO) release, which is needed for suppression of smooth muscle cells (SMCs) and proliferation of endothelial cells (ECs). The characterization of the film surface was conducted via various analyses. Through the surface modification of PLLA films, they have multifunctional abilities to overcome problems of BVS effectively such as X-ray penetrability, inflammation, thrombosis, and neointimal hyperplasia. These results suggest that the modification of biodegradable PLLA using TIBA, MH, PDA, and Cys will have important potential in implant applications.

## 1. Introduction

Biodegradable vascular scaffold (BVS) has been suggested to alternate problems associated with drug-eluting stent (DES). The DES delays stent endothelization as well as hypersensitivity reactions, but the BVS prevents the lumen expansion associated with late favorable remodeling, reduces restenosis rates, the need for repeat revascularization, and complete degradation [1]. Because of these advantages, BVS can be a new future direction of coronary stents with biocompatibility and biodegradability compared with previous generations. However, BVS has several problems that can make an acidic environment from by-product, hard to find after implantation, and obstruct vessels. As a result, severe inflammation, thrombosis, and in-stent restenosis can occur after coronary intervention by using commercially available biodegradable stents [2,3]. For enhancing re-endothelialization and suppressing in-stent restenosis, the regeneration behavior of endothelial cells has been studied in various aspects [4,5]. In the approach to developing such problems of BVS, we have selected some novel materials.

When BVS was implanted in the body, polymers such as poly(L-lactic acid) (PLLA) are not easy to find on X-ray computed tomography (CT) due to weak radiopacity. Consequently, the lack of radiopacity of BVS can be a severe problem compared with DES. To solve this problem, BVS could be contained materials that have high X-ray opacity. Among them, Iodine has a radiopacity because of its high atomic weight (standard atomic weight = 126.9), therefore, iodine-containing compounds have been adopted with polymers for intervention [6,7]. We fabricated a system coating 2,3,5-triiodobenzoic acid (TIBA) with poly(D,L-lactic acid) (PDLLA). The PDLLA which was known as a biodegradable polymer for using on surface coating agent degrades slower than poly(lactic-co-glycolic acid) (PLGA) and has higher mechanical properties than PLGA [8]. However, compared with PLLA, PDLLA was degrades rapidly and caused inflammatory response by acidic byproducts of PDLLA. In this study, we added magnesium hydroxide [Mg(OH)_2_, MH] in the coating solution for neutralizing acidified bloodstream owing to the degradation of PDLLA [9,10]. As a result, modified BVS has radiopacity to trace CT image and anti-inflammation function through pH neutralization.

Nitric oxide (NO) was continuously secreted by healthy vascular endothelial cells (ECs), maintaining vascular tone as the ground state and the release of NO plays an important role in maintaining homeostasis and the blood vessels [11]. Furthermore, it obstructs the absorption of platelet and monocytes in the vascular wall, suppresses the proliferation of smooth muscle cells (SMCs), and stimulates the proliferation of ECs [12,13]. However, when BVS was implanted in the body, wounded endothelial cells reduce to release NO, then it causes hyperplasia of SMCs, and restriction of the proliferation of ECs results in thrombosis. The importance of the continuous release of NO is evident. We fabricated a modified PLLA that can generate NO by coating polydopamine (PDA) and then cystamine (Cys) on the PLLA surface. Of all the surface modification methods, the polydopamine coating has been widely applied from inspired by the co-existence of catechol and amine, which are mussel adhesive proteins [14]. For inducing NO release, glutathione (GSH) and S-nitroso-N-acetyl-D,L-penicillamine (SNAP) are needed, and GSH links to Cys as reacting with disulfide bond of Cys. After then linked GSH attacks NO bond of SNAP which is NO donor, eventually, it releases NO [15,16].

For applying the above materials on BVS, we fabricated PLLA films as the surface of BVS and divided PLLA films into two coating layers. In the first layer, PLLA film was coated by TIBA and MH with PDLLA for radiopaque and neutralization of the acidic environment. Moreover, to prevent toxicity from TIBA, we put additionly PDLLA on the first layer for obstructing the burst release of TIBA for protecting cells. In sequence, the second layer of coated PLLA films is composed of PDA and then Cys for the generation of NO release, which is needed for suppression of smooth muscle cells and proliferation of endothelial cells.

The aim of this study was to make and characterize surface-modified PLLA films with multifunctional abilities to overcome previous problems of BVS effectively such as penetrability, inflammation, thrombosis, and neointimal hyperplasia.

## 2. Materials and Methods

### 2.1. Materials

Poly(L-lactic acid) (PLLA, Mw = 200 kDa) and poly(D,L-lactic acid) (PDLLA, R205S, Mw = 10–18 kDa) were purchased from Evonik Industries AG (Essen, Germany). The 2,3,5-triiodobenzoic acid (TIBA, 98%), magnesium hydroxide (MH, 99.0%), dopamine hydrochloride, cystamine dihydrochloride (96%), L-glutathione reduced (98.0%), tris(hydroxymethyl)aminomethane(ACS reagent, 99.8%), hexamethyldisilazane (HMDS, 99%), S-nitroso-N-acetyl-D,L-penicillamine (97%) and glutaraldehyde (GA) solution (25%) were purchased from Sigma-Aldrich (St. Louis, MO, USA). The tetrahydrofuran (THF, 99.8%), 1,4-dioxane (DO, 99.5%), and 1N-sodium hydroxide standard solution were purchased from Daejung Co. Ltd. (Seoul, Korea). The ethanol, methanol, acetonitrile, water (HPLC grade) was purchased from J.T. Baker (Phillipsburg, NJ, USA) and phosphate-buffered saline (PBS) solution was purchased from Hyclone in GE Healthcare Life Sciences (Seoul, Korea). A cell counting kit (CCK-8) was purchased from Dongin LS (Seoul, Korea). Human coronary artery endothelial cells (HCAECs), human coronary artery smooth muscle cells (HCASMCs), endothelial cell growth medium-2 (EGM-2), and smooth muscle cell growth medium (SmGM) were purchased from Lonza (Basel, Switzerland). IL-6 and IL-8 enzyme-linked immunosorbent assay (ELISA) kits were purchased from R&D Systems (Minneapolis, MN, USA).

### 2.2. Preparation of Multifunctional PLLA Films

#### 2.2.1. The First Coating Layer: TIBA/MH Coating on PLLA Films

PLLA films were prepared from chloroform solution and cut in size at 0.5 cm × 1 cm. For the preparation of coating solution, THF/DO which is co-solvent was made by mixing THF and DO with 90:10 (v/v%). TIBA and PDLLA was dissolved in THF/DO solvent at a concentration of 10 mg/mL, 0.2% each. After then, MH (20 phr of PDLLA) was dissolved into THF/DO solution. The PLLA films were coated by an ultrasonic spray coater from Noanix (Cheongju, Korea). The coating process was conducted at 30–40% of humidity [17].

#### 2.2.2. The Second Coating Layer: PDA/Cys Coating on PLLA Films

Before the coating process, the coated radiopaque films were hydrated in ethanol (10%) for 5 min. After then, the films were immersed into the prepared dopamine solution (1 mg of dopamine and l mL of 10 mM Tris solution (pH 8.5), then 1 and 10 mM Cys solutions were added and allowed to react at 100 rpm shacking condition at room temperature for 24 h. The PDA/Cys coated films were washed three times with distilled water to remove residual dopamine [18].

### 2.3. Characterization of Coated PLLA Films

#### 2.3.1. X-Ray Images of Radiopaque PLLA Films

X-ray scanning was conducted by portable digital fluoroscopy (SX-DRF0815M, NanoFocusRay, Iksan, Korea) with a complementary metal oxide semiconductor (CMOS) flat-panel detector was used. The X-ray source was generated with acceleration voltage at 75 kV, electric current at 0.18 mA, and 15 fps of frame rate. The system was offered both a fluoroscopic imaging mode and a radiographic imaging mode. The prepared films were coated by 10 mg/mL of TIBA solution with different volume (0.2, 0.4, 0.6, and 0.8 mL). Bare metal stent (BMS) which is composed of cobalt-chrome, was used as a positive control and PLLA film was used as a negative control.

#### 2.3.2. Quantitative Analysis of MH

Quantitative analysis of the amount of MH on coated PLLA films was conducted by Inductively Coupled Plasma-Optical Emission Spectrometer (ICP-OES, Optima 7300 DV, PerkinElmer, Shelton, CT, USA) which set up with a flow rate at 0.8 L/min. For conducting ICP-OES, the films were melted in 5% nitric acid at 60 °C for 2 h and magnesium standard was prepared. All samples were conducted in three replicates.

#### 2.3.3. Water Contact Angle

To evaluate the hydrophilicity of the coating surface layer, the angle of the water droplet on the PLLA films was measured by optical bench-type contact angle measurement (Phoenix 300, Surface Electro Optics, Suwon, Korea). The deionized water (20 μL) was dropped on the different surface regions of the PLLA film and calculated the average of right and left angle within 10 s by 10 times. All samples were conducted in three replicates.

#### 2.3.4. NO Release Analysis

To demonstrate the NO release, 4-amino-5-methylamino-2,7-difluororescein (DAF-FM, Sigma-Aldrich, St. Louis, MO, USA) was used to detect and quantify the low concentration of released NO. It is essentially nonfluorescent until it reacts with NO to form a fluorescent benzotriazole. Ten μM of GSH/SNAP and DAF-FM solution diluted in DMSO were prepared. The coated films were put on a 24-well plate and each 200 mL of GSH/SNAP and DAF-FM solution was added and then incubated for 40 min at 37 °C. After that, the films were removed, and remained solutions were measured by a microplate reader (SpectraMax M2, Molecular Devices, San Jose, CA, USA) set up with 495 nm of UV wavelength.

#### 2.3.5. Elemental Composition and Chemical Bonding Analysis

The surface elemental composition analysis of coated films was conducted by X-ray photoelectron spectroscopy (XPS, Nexsa, ThermoFisher Scientific, Waltham, MA, USA). For XPS condition, microfocus monochromatic X-ray (Al-Kα (1486.6 eV) was used as a source, Adventitious Carbon (284.8 eV) as charging correction, 400 μm × 400 μm as beam spot size, and Ar ion + electrons as a dual neutralizer. Chemical bonding of the film surface was measured by attenuated total reflection-Fourier transform infrared spectroscopy (ATR-FTIR, Spectrum 100, Perkin Elmer, Waltham, MA, USA) in the range of 800–4000 cm^−1^.

#### 2.3.6. Surface Morphology

Surface morphology of the coated PLLA films was observed by scanning electron microscope (SEM, SNE-3200M, SEC Co., Suwon, Korea) which set up with acceleration voltage at 5 kV, emission current at 100 uA, and magnification at ×1000. The prepared films were TIBA/MH coated, top coating on TIBA/MH, PDA/Cys coating on top coated films, and PLLA film as a control. Before observation, the films were sputter-coated with gold for 1 min.

#### 2.3.7. Degradation Behavior

Each film was weighed (initial weight (W_0_) and placed in 10 mL vials with 2 mL of PBS solution, then put in the water bath at 37 °C which is known as physiological temperature. pH level was measured via pH-meter (Mettler Toledo, Zurich, Switzerland), and final residual weight (W_t_) is measured at 1, 3, 5, 7, 14, 21, and 28 days, washed by deionized water, and dried under vacuum oven for 3 h. The percentage of residual weight was calculated from the following equation:

The percentage of degradation (%) = [(W_0_ − W_t_)/W_0_] × 100.

After measuring pH, 2 mL of PBS solution were extracted from vials and filled new 2 mL of PBS solution. After putting again in the water bath, extracted PBS solution was freeze-dried at −70 °C for 24 h. Remained residues were analyzed by high-performance liquid chromatography (HPLC, Agilent, Santa Clara, CA, USA) set up with 234 nm of UV wavelength, 1 mL/min of flow rate for 10 min, and using a mobile phase with 45% of methanol, 40% of acetonitrile, and 15% of water [19].

### 2.4. In Vitro Study

#### 2.4.1. Cell Culture

HCAECs (P7) and HCASMCs (P7) were sowed with a density of 1 × 10^6^ cells/mL and grown in a T75 tissue culture flask with 13 mL of EGM-2 and SmGM, respectively. HCAECs were incubated in a humidified atmosphere incubator with 5% CO_2_ at 37 °C. When cells reached more than 80% confluence, the cells were detached by 3 mL of 0.125% of trypsin/EDTA solution for 3 min.

#### 2.4.2. Cell Proliferation Assay

The prepared films were placed in 24-well plates, sterilized on UV light of a clean bench for 30 min, and hydrated with 10% ethanol for 10 min. Fifty μL of 1 × 10^4^ cells/mL were seeded on each film and took adhesion time for 3 h, after then 950 μL of the medium were added to each film. In total, 10 μM of GSH/SNAP were injected per 6 h for 24 h. After 24 h, remained media were removed, 400 μL of 10% CCK-8 solution was added to each well in dark condition and incubated for 2 h. After incubating, 100 μL of CCK-8 solution transferred to a 96-well plate and measured by a microplate reader set up with 450 nm of UV wavelength [20].

#### 2.4.3. Inflammation Response Analysis

To evaluate cytokine levels of HCAECs, IL-6 and IL-8 were measured using ELISA assay kit. The process was followed by ELISA assay protocol. In brief, ELISA was accomplished in a 96-well plate and the samples were incubated at room temperature for 2 h. After aspirating the samples, each well was rinsed with a washing buffer. The conjugate solution was put to each well and incubated for 2 h at room temperature. Additionally, then, the substrate solution was added to each well. The final step was the addition of the stop solution to each well. When the enzyme reaction was completed within 30 min, the plate was placed into a plate reader and the optical density was detected for each well at 450 nm.

#### 2.4.4. Platelet Adhesion Test

The films were sterilized under UV light for 30 min and hydrated in 1 mL of PBS solution for 1 h. The pre-wetted films were placed in the 24-well plates, the 1 mL of the human platelet solution (2 × 10^7^ platelets/mL) was added, and the films were incubated for 2 h at 37 °C. After then, the films were washed out with PBS solution 3 times. For observing SEM images of adhered platelets, the samples were fixed by diluted GA solution (2.5%) for 1 h and then dehydrated with each 50, 60, 70, 80, 90, and 100% of ethanol solutions. The dehydrated samples were coated with the HMDS/ethanol (2:1, 1:1, and 1:2) solutions each for 10 min, and finally coated with gold. Meanwhile, adhered platelets were lysed by 0.5 mL of 2% Triton X-100 for 15 min and the lysed solution was analyzed by lactose dehydrogenase (LDH) assay. Then, the LDH assay was conducted according to the manufacturer’s instructions (MK401, Takara, Kusatsu, Japan). The absorbance was investigated at 490 nm using a SpectraMax M2 plate reader (Molecular Devices, San Jose, CA, USA), and a standard curve was applied to determine the total number of platelets attached to the various film types [21].

### 2.5. Statistical Analysis

All statistical analysis was accomplished using GraphPad Prism (San Diego, CA, USA). One-way ANOVA with Tukey’s multiple comparison posttest was performed to compare the samples. The results considered no significant (ns) when *p* > 0.05 and statistically significance when * *p* < 0.05, ** *p* < 0.01, and *** *p* < 0.001, and **** *p* < 0.0001.

## 3. Results & Discussion

### 3.1. Preparation and Characterization of Radiopaque and NO Released Film

**Scheme 1:**
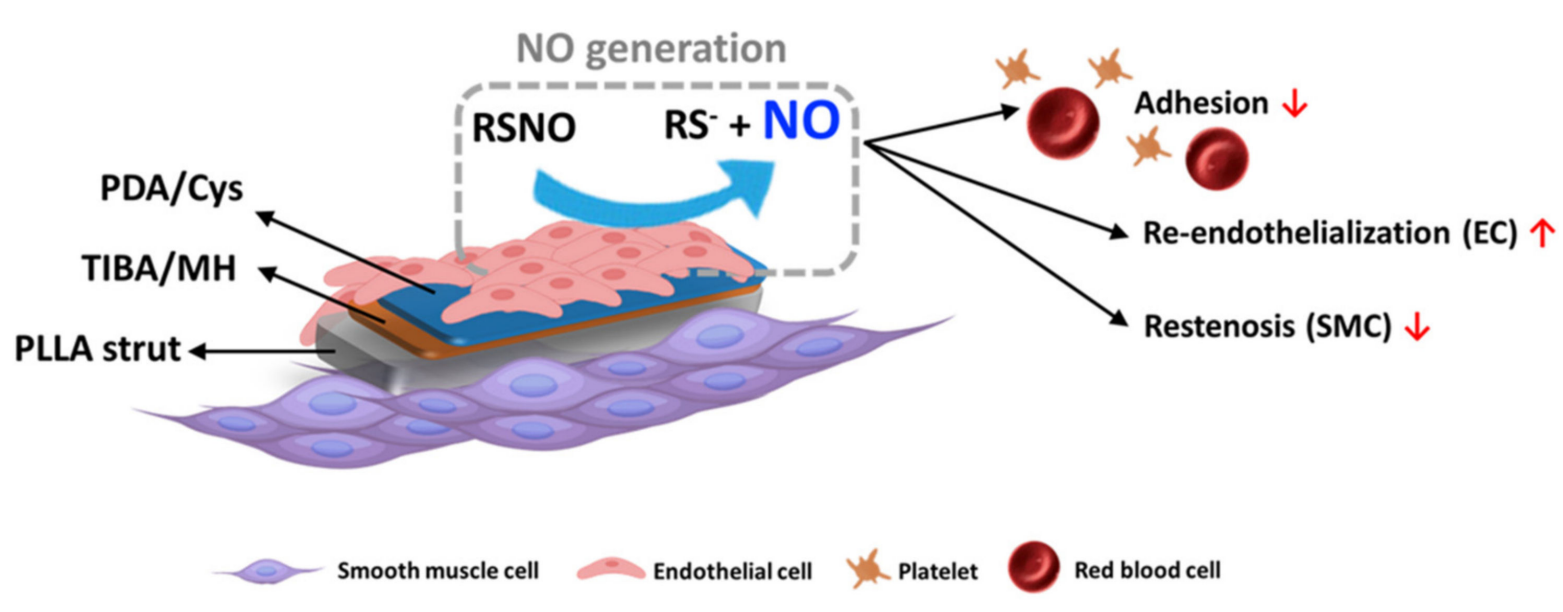
The coating layer of a multifunctional biodegradable stent strut film (1st layer: TIBA/MH and 2nd layer: PDA/Cys); the enhancement of re-endothelization by ECs adhesion and the suppression of SMCs and platelet adhesion by NO release.

Before preparing the radiopaque and NO released film, PLLA was chosen as a biomaterial of the film, which is widely used in the composition of BVS. The bare PLLA film has not a radiopaque, then it was not able to find the X-ray image. To solve this problem, we coated radiopaque material, TIBA with PDLLA as coating polymer, and also MH as additive. MH can neutralize the acidic environment caused by late PLLA degradation and early release of PDLLA and TIBA. After coating the first layer, for inducing NO release, PDA with Cys was coated with the deep coating method. Eventually, there are two layers of PLLA films that have the ability of radiopaque and NO release. Releasing NO results in re-endothelialization of HCAECs, suppression of SMCs, and reducement of platelet adhesion (Scheme 1).

**Figure 1:**
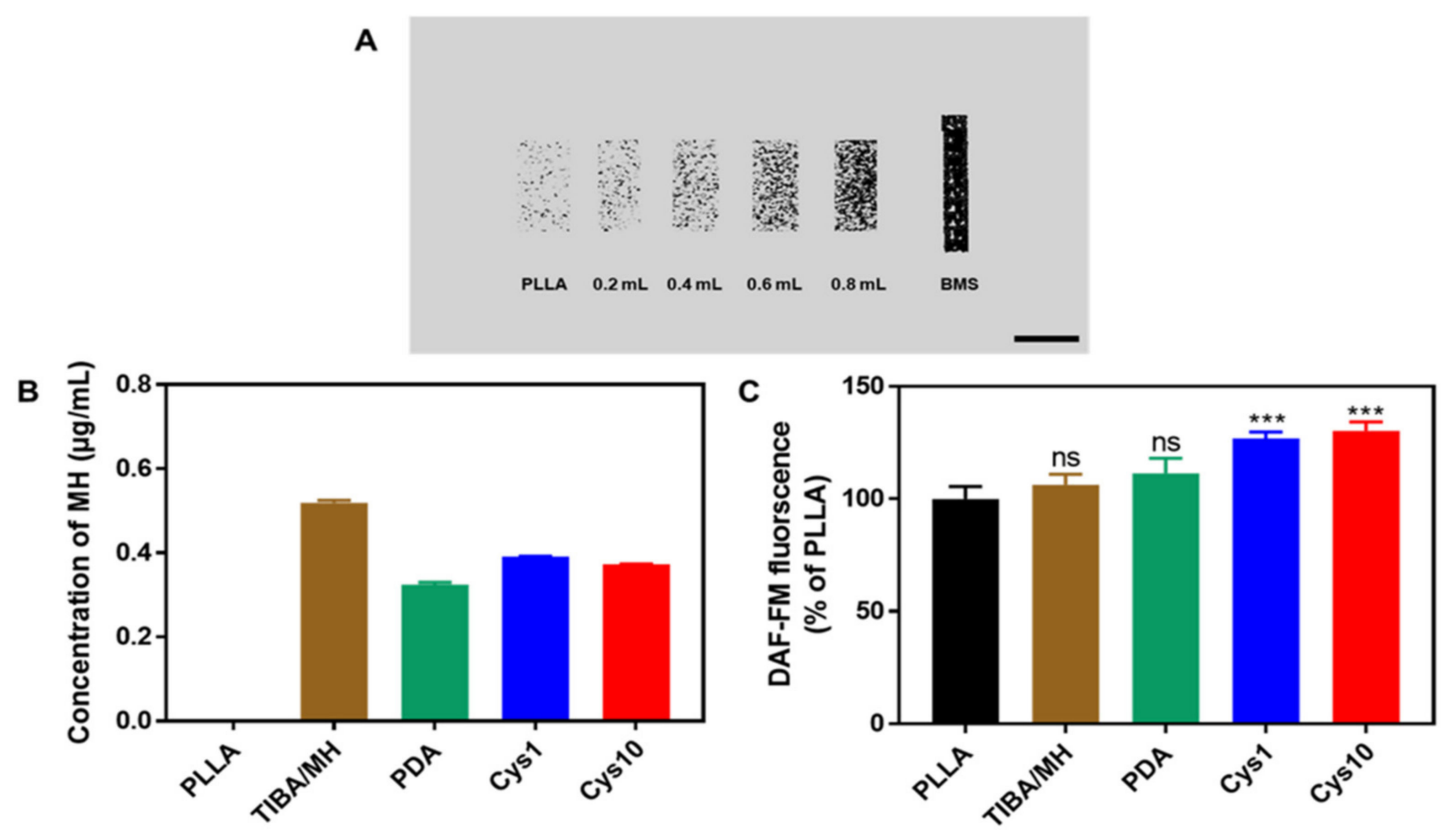
(**A**) X-ray images of PLLA films coated with various concentrations of TIBA; The positive control corresponds to cobalt-chrome BMS (Scale bar = 10 mm), (**B**) ICP-OES for measuring MH contents, and (**C**) DAF-FM fluorescence ratio to PLLA (%) for NO release (*** *p* < 0.001 vs. PLLA).

To identify radiopaque of the films, the different volume of TIBA solution (10 mg/mL) was coated (Figure 1A). The coating with 0.8 mL of TIBA solution could almost reach radiopaque ability of cobalt-chrome BMS as positive control on the X-ray image, then 0.8 mL of TIBA solution was chosen as coating condition. After coating PDA/Cys, the amount of coated magnesium was measured by ICP-OES (Figure 1B). It indicated that magnesium was detected on each coated film except PLLA film and still remained after coating PDA/Cys on film. When comparing PDA and PDA/Cys, TIBA/MH displayed the increased magnesium portions compared to others. It considered that a little amount of magnesium was leaked slightly after PDA/Cys coating, but there was not different significantly compared with TIBA/MH. To evaluate NO release by reaction with Cys and GSH/GSNO, DAF-FM fluorescence was conducted (Figure 1C). In the group of Cys1 and Cys10 could detect a higher intensity of DAF-FM fluorescence compared with PLLA, TIBA/MH, and PDA. This result reveals that Cys on coated films releases NO by reaction with GSH/SNAP.

**Figure 2:**
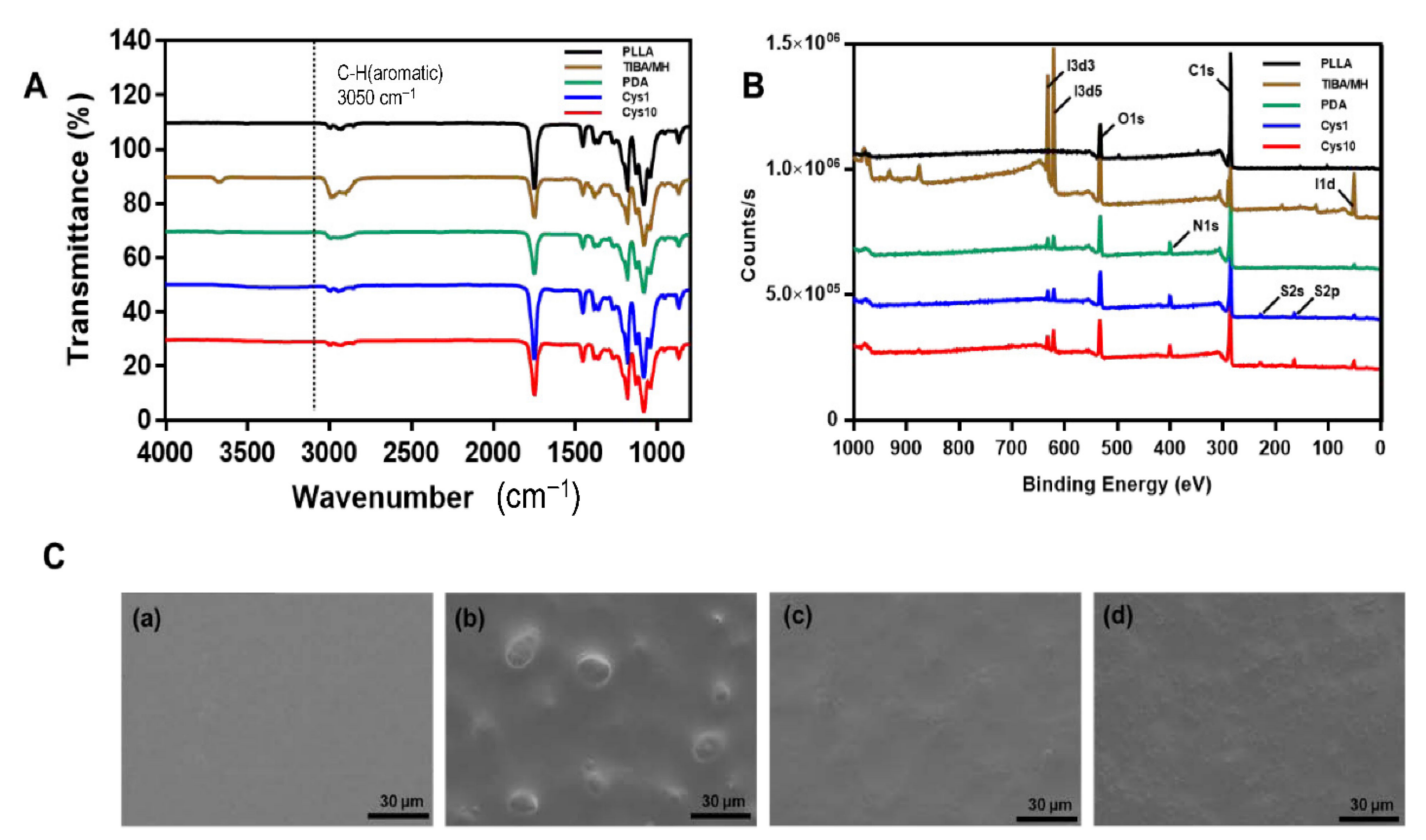
(**A**) ATR-FTIR spectra, (**B**) XPS spectra of of PLLA, TIBA/MH, PDA, Cys1, and Cys10, and (**C**) Surface morphology of (**a**) PLLA, (**b**) TIBA/MH, (**c**) PDLLA top coated TIBA/MH, and (**d**) PDA/Cys coated films (Scale bar = 30 μm).

The ATF-FTIR spectrum of PDA/Cys was investigated in comparison with PLLA, TIBA/MH, and PDA (Figure 2A). The spectra of all samples were similar to that of PLLA, but the TIBA/MH spectrum contained –OH stretching vibration at 3700 cm^−1^ due to MH and C-H stretching vibration of aromatic compounds at 3050 cm^−1^ on the surface of coated TIBA [22]. These peaks could not be confirmed on the PDA and PDA/Cys film since it was after the second coating. In XPS spectra (Table 1), TIBA has the highest ratio of iodine composition (27.69%), then after PDA/Cys coating, the ratio of iodine decreased because of the coverage of the second coating layer, and both nitrogen and sulfur portions were detected, but the sulfate portion cannot be detected on only PDA coated films. Figure 2B was observed for high spectra of I3d3, I3d5, and I4d from the first layer of the films (TIBA/MH), and N1s, S2s, and S2p were presented at PDA/Cys coated film [23]. As the result, these peaks were observed for elemental composition of each coating layer and demonstrated that the desired PLLA film samples were prepared. Figure 2C confirmed the surface morphology of each coated film. It shows PLLA film has a smooth surface (a), but after coating TIBA/MH, the surface of the film became rough due to the precipitation of TIBA. To solve this problem, the PDLLA solution was used to coat on the top of the rough TIBA/MH layer. Then, the result of surface morphology indicated much smoother (c). The surface (d) after PDA/Cys coating exhibited similar surface morphology compared with (c) surface. In addition, the water contact angle was measured to compare the surface wettability. The PLLA, Cys1, and Cys10 films have 62.16, 55.83, and 54.08°, respectively (Table 1). It implies that PDA and Cys films displayed a lower angle than PLLA film, meaning that PDA and Cys films have more hydrophilic surface than PLLA film [24].

### 3.2. Degradation Behavior of the Films and Cumulative Release of TIBA

**Figure 3:**
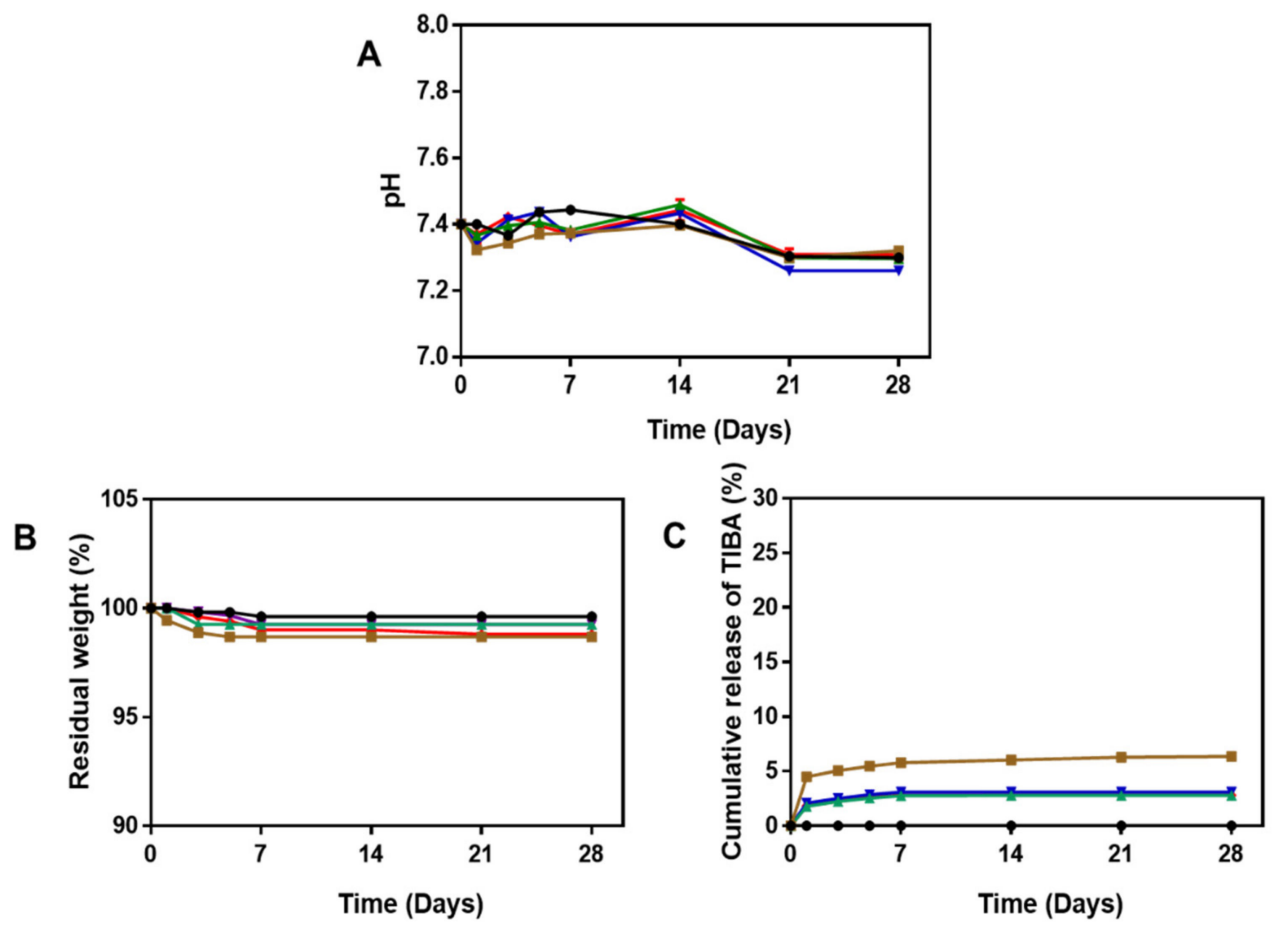
(**A**) The changes in pH, (**B**) residual weight (%), and (**C**) cumulative release of TIBA of the coated PLLA films during degradation in PBS solution at 37 °C for 28 days, there was no significant difference between five groups. (PLLA = 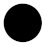, TIBA/MH = 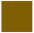, PDA = 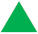, Cys1 = 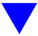, and Cys10 = 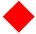).

The degradation behavior of the coated PLLA films was investigated in pH 7.4 at 37 °C for 28 days (Figure 3). The pH of all samples was not changed for 28 days among pH 7.2–7.4 (a). Residual weight of all samples was nearly not changed as well (b), which indicates the PLLA films maintain stability under physical condition for 28 days. The cumulative release of TIBA (c) shows that the TIBA/MH film was released more than 5% of the total amount of coated TIBA, but after covering the second layer, TIBA was released less than 3% as in PDA, Cys1, and Cys10 films for 28 days. It suggests that due to less release of TIBA for 28 days, the coated films have a radiopaque continuously but have not to effect on cell toxicity.

### 3.3. In Vitro Cell Proliferation

**Figure 4:**
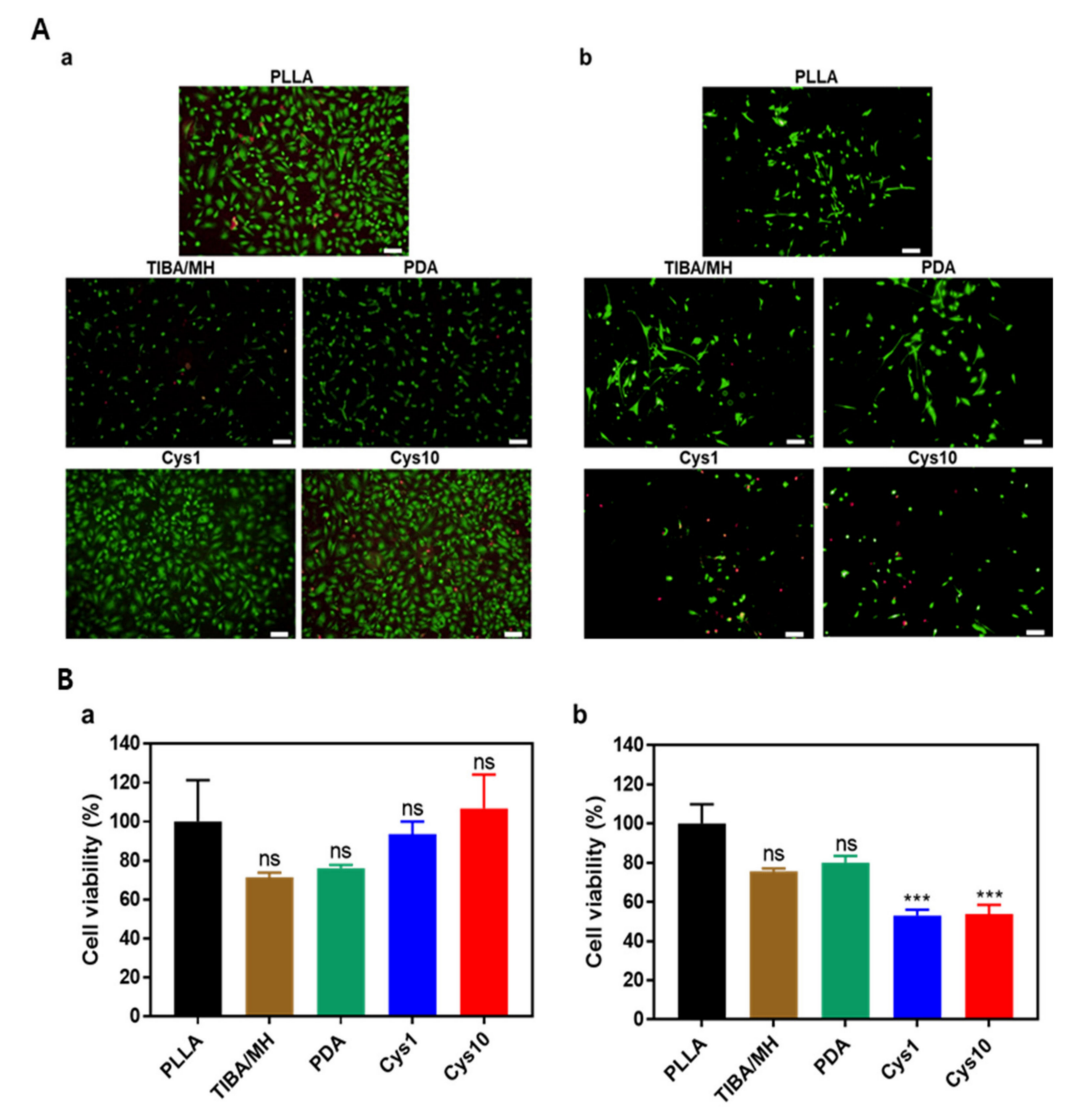
(**A**) Live/dead assay of (**a**) HCAECs and (**b**) HCASMCs with SNAP (Live cells: green and dead cells: red, scale bar = 100 μm) and (**B**) Cell viability of (**a**) HCAECs and (**b**) HCASMCs on the coated films with SNAP for 24 h (*** *p* < 0.001 vs. PLLA).

Cell proliferation of HCAECs and HCASMCs on the PLLA, TIBA/MH, and PDA/Cys films was determined for 24 h (Figure 4A,B). HCAECs were well attached on Cys1 and Cys10 compared with other samples. However, the cell proliferation on Cys1 and Cys10 films significantly (*** *p* < 0.001) decreased compared to PLLA. Due to NO release, the proliferation of HCAECs increased, whereas the proliferation of HCASMCs was suppressed. The calcein AM staining shows fully compact live HCAECs on Cys1 and Cys10. On the other hand, HCASMCs on Cys1 and Cys10 were changed to a more round shape and stained less than other live cells of samples, which means that the NO release induced proliferation of HCAECs and suppressed adhesion of HCASMCs. The NO release has a significant influence on the inhibition of HCASMCs migration and promotion HCAECs proliferation, as well as causing smooth muscle relaxation [25,26,27]. The decreased NO production has been related to endothelial malfunction. The NO release from the endothelium has been supposed to be responsible for HCASMCs relaxation that leads to alter in vasodilatation and shear stress, while the former alters the morphology of HCAECs and the latter is related to HCASMCs.

### 3.4. Inflammation Assay

**Figure 5:**
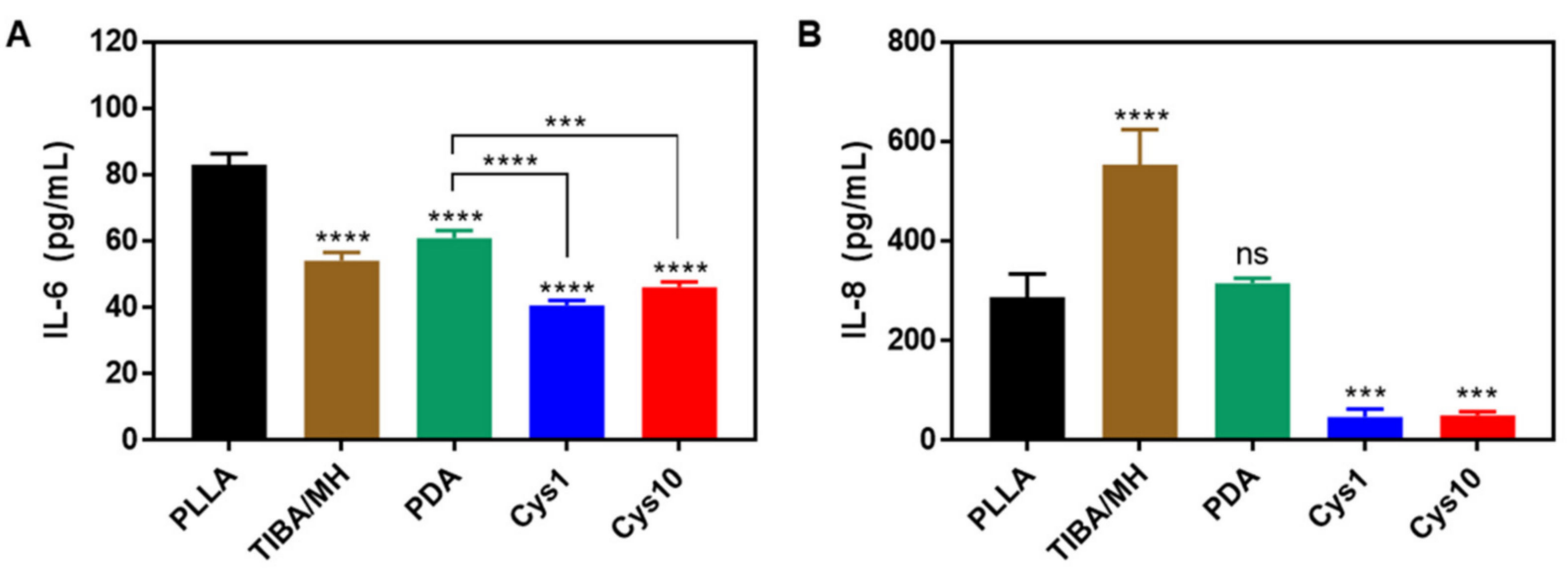
The expression levels of cytokine factor (**A**) IL-6 and (**B**) IL-8 in the proliferation of HCAECs on the coated films for 24 h (*** *p* < 0.001, **** *p* < 0.0001 vs. PLLA).

The inflammation of HCAECs was analyzed by IL-6 and IL-8 ELISA. The expression levels of IL-6 in TIBA/MH, PDA, Cys1, and Cys10 was significantly (**** *p* < 0.0001) lower than PLLA, especially in Cys1 and Cys10 groups, whereas their expression levels of IL-6 were reduced (*** *p* < 0.001) compared with PDA (Figure 5A). The expression level of IL-8 in TIBA/MH was higher (**** *p* < 0.0001) than that of PLLA (Figure 5B). It seems that the release of acidic compounds of TIBA in the early phase increased the IL-8 level. However, in Cys1 and Cys10 groups, their IL-8 levels were lower (*** *p* < 0.001) than that of PLLA. These results could prove the effect of MH. The addition of MH in the coating process was demonstrated to reduce the level of cytokines. MH can neutralize the acidic byproducts of PDLLA, then suppress inflammation and cytotoxicity [28,29]. In addition, The NO release could also provide inhibiting inflammation, so that the Cys1 and Cys10 groups were significantly lower than other groups in IL-6 and IL-8 [30].

### 3.5. Platelet Adhesion and Activation

**Figure 6:**
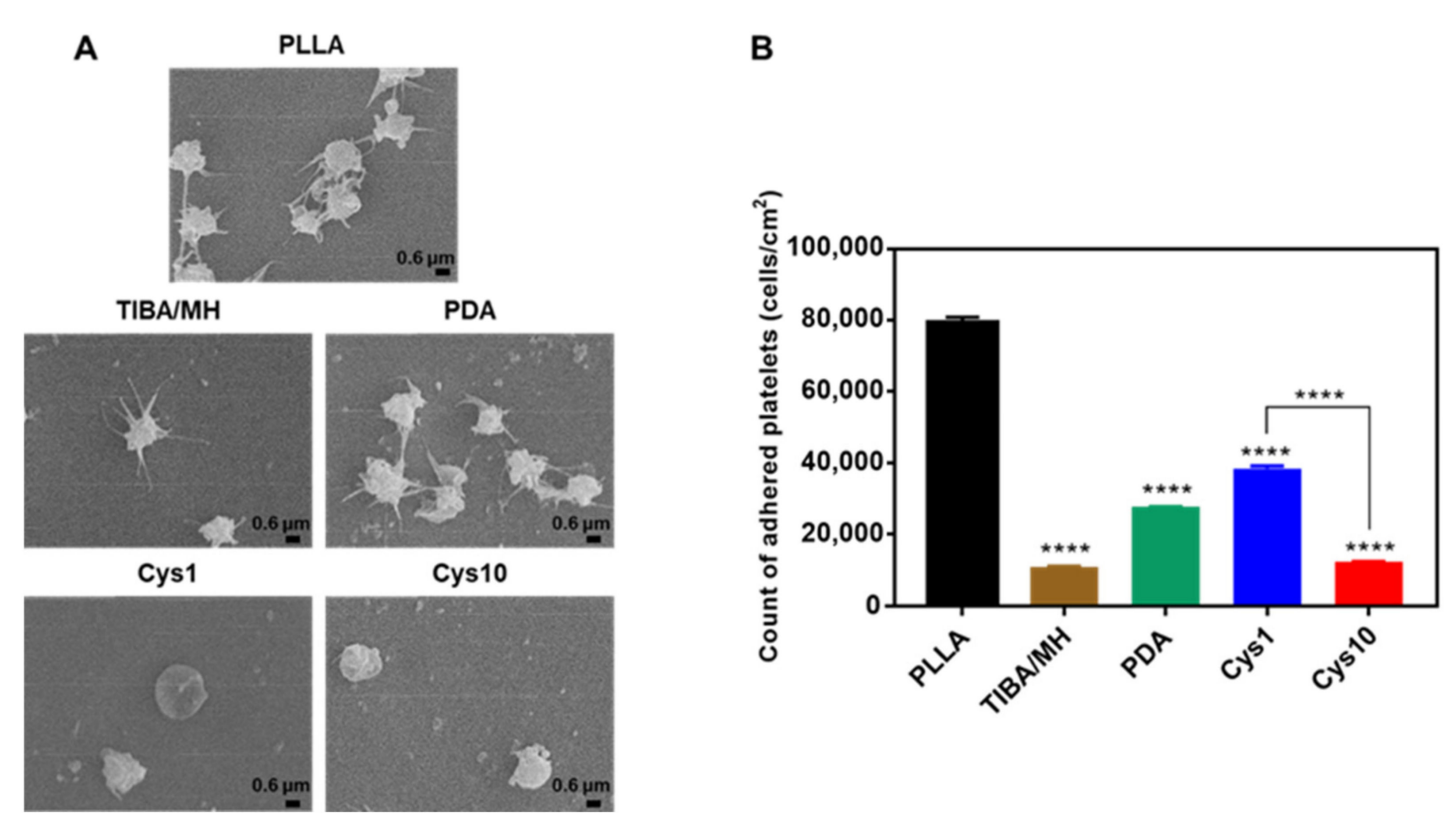
(**A**) SEM images (Scale bar = 1 μm) and (**B**) LDH assay of adhered platelets on the surface of the coated films (**** *p* < 0.0001 vs. PLLA).

The morphology of adhered platelets on the surface of films was observed by SEM (Figure 6A). The adhered platelets on the surface of PLLA, TIBA/MH, and PDA have a spread of dendritic morphologies, which means activated. Especially, the surface of PLLA and PDA could be observed that the aggregation and activation of adhered platelets by crosslinking was progressed. Meanwhile, the morphologies of adhered platelets on Cys1 and Cys10 surfaces were almost round shape. Moreover, the quantitative analysis of adhered platelets was conducted by LDH assay (Figure 6B). TIBA/MH, Cys1, and Cys10 have significantly (**** *p* < 0.0001) lower adhered platelets than PLLA. Compared with TIBA/MH, PDA and Cys1 have relatively high adhered platelets. It seemed that the cytotoxicity of TIBA obstructed platelet adhesion on the surface of TIBA/MH, and cell-adhesive PDA and relatively lower NO release in Cys1, respectively could induce to adhere the platelets on their surfaces. However, Cys10 has significantly lower adhered platelets than Cys1. These results can prove that Cys10 has the ability to anti-platelet adhesion and activation [21,31,32].

## 4. Conclusions

The surface modification of PLLA with TIBA, PDA, and Cys was successfully accomplished and significantly improved in NO generation from S-nitrosothiols (RSNO) in the presence of SNAP. The ECs were enhanced and SMCs and platelet adhesion were suppressed from the NO release on the film. Additionally, the addition of TIBA increased the X-ray opacity and the incorporation of MH into PLLA synergistically reduced the acid-induced inflammation because of the pH neutralization of PLLA acidic degradation by basic inorganic MH. Such a multifunctional biodegradable vascular polymer scaffold not only enhances the X-ray opacity and re-endothelialization but also reduces inflammation and SMCs restenosis by coating biocompatible additives. Therefore, it is suggested that surface-modified PLLA with TIBA/MH and PDA/Cys would be promising as a novel biomaterial for biodegradable implants including vascular stents. In the future, we expect that it can be possible to fabricate BVS from this multi-functional PLLA film and then well-made new version of BVS will be applied to in vivo animal study.

## Figures and Tables

**Table 1:** Surface characterization of the coated PLLA films.

Sample	Mg (ppm) ^a^	Atomic Composition (%) ^b^					WCA (θ) ^c^
		C	O	I	N	S	
PLLA	-	87.24	12.76	-	-	-	62.16 ± 1.42
TIBA	519.03 ± 5.91	48.01	24.3	27.69	-	-	59.06 ± 2.22
PDA	324.08 ± 5.73	77.09	15.62	1.42	5.88	-	55.18 ± 1.04
Cys1	391.26 ± 0.68	74.11	15.67	1.27	6.82	2.14	56.62 ± 1.31
Cys10	373.06 ± 0.72	71	16.43	2.5	6.46	3.61	54.08 ± 2.66

^a^ Mg components from ICP, ^b^ atomic compositions from XPS, and ^c^ water contact angle.

## Data Availability

The data presented in this study are available in this study. Additional information could be available on request from the corresponding author.
